# Individual Differences in Spontaneous Expressive Suppression Predict Amygdala Responses to Fearful Stimuli: The Role of Suppression Priming

**DOI:** 10.3389/fpsyg.2017.00001

**Published:** 2017-01-31

**Authors:** Shengdong Chen, Zhongyan Deng, Yin Xu, Quanshan Long, Jiemin Yang, Jiajin Yuan

**Affiliations:** ^1^The Laboratory for Affect Cognition and Regulation (ACRLAB), Key Laboratory of Cognition and Personality of Ministry of Education (SWU), Faculty of Psychology, Southwest UniversityChongqing, China; ^2^Department of Youth Education and Management, Beijing Youth Politics CollegeBeijing, China

**Keywords:** spontaneous emotion regulation, expressive suppression, amygdala, fMRI, fear

## Abstract

Though the spontaneous emotion regulation has received long discussions, few studies have explored the regulatory effects of spontaneous expressive suppression in neural activations, especially in collectivistic cultural context. The functional magnetic resonance imaging (fMRI) study aimed to examine whether individual differences in the tendency to use suppression are correlated with amygdala responses to negative situations when individuals are unconsciously primed with expressive suppression. Twenty-three healthy Chinese undergraduates completed an fMRI paradigm involving fear processing, and a synonym matching task was added to prime participants with the unconscious (automatic) expressive suppression goal. Participants completed measures of typical emotion regulation use (reappraisal and suppression), trait anxiety, and neuroticism. Results indicated that only in emotion suppression prime condition, greater use of suppression in everyday life was related to decreased amygdala activity. These associations were not attributable to variation in trait anxiety, neuroticism, or the habitual use of reappraisal. These findings suggest that in collectivistic cultural settings, individual differences in expressive suppression do not alter fear-related neural activation during suppression-irrelevant context. However, unconscious suppression priming facilitates the manifestation of individual differences in the neural consequence of expressive suppression, as reflected by the priming-specific decrease of emotional subcortical activations with more use of expressive suppression.

## Introduction

It is widely acknowledged that individuals differ systematically in their habitual use of emotion regulation strategies (Gross and John, 2003; John and Gross, 2004). For instance, those who have a high tendency to alter the meaning of a potentially emotion-eliciting situation in order to change its emotional impact as reappraisers were called as “reappraisers” (Gross and John, 2003). Increasing evidence supports that the habitual use of emotion regulation—whether assessed with retrospective self-reports or questionnaires—can be regarded as an index of spontaneous emotion regulation (Egloff et al., 2006; Drabant et al., 2009; Gyurak et al., 2011). Spontaneous emotion regulation refers to that absent of explicit instructions, individuals use certain regulation strategies spontaneously (automatically) that best fit their personal preference in a given emotional situation, in the absence of explicit instructions (Egloff et al., 2006; Gyurak et al., 2011). Generally, if individuals self-report higher habitual use of certain emotion regulation strategies, they are more likely to use such strategies to regulate their emotion responses to emotional stimuli or events automatically (Drabant et al., 2009; Gyurak et al., 2011).

Previous studies of spontaneous (automatic) emotion regulation have mainly adopted an individual-difference approach to study its implications for many domains of life (Gross and John, 2003; Egloff et al., 2006; Drabant et al., 2009). Specifically, in contrast to the experimental approach, the use of certain regulations strategies (if any) are not induced by external instructions during spontaneous regulation. Instead, participants are free to use the strategy according to their personal preference. And the extent to which participants spontaneously use suppression and/or reappraisal during the task are assessed by retrospective self-reports or questionnaires. Gross and John (2003) developed reliable brief trait measures of suppression and reappraisal. They found that in terms of relationships with other variables, the pattern of results is similar to that reported for the experimental approach: the habitual use of reappraisal was associated with less negative affect, better interpersonal functioning, and well-being. By contrast, the habitual use of suppression correlated with a less beneficial profile of emotional functioning. Egloff et al. (2006) examined the associations of spontaneous emotion regulation with experiential and physiological emotion responding during evaluated speaking tasks. The pattern of their results was also similar to that reported for the experimental approach: suppression was associated with less anxiety expression, greater physiological responding, reduced memory for the speech but had no impact on negative affect. By contrast, reappraisal has no impact on physiology and memory, but led to less expression and affect. More recently, an functional magnetic resonance imaging (fMRI) study confirmed that participants with higher reported use of reappraisal were more likely to engage in spontaneous reappraisal, and showed decreased amygdala activity during the processing of emotionally negative facial expressions (Drabant et al., 2009).

However, currently little is known about the regulatory effects of spontaneous expressive suppression in neural activations, especially in collectivistic cultural context. Expressive suppression is a form of response modulation that involves inhibiting ongoing emotion-expressive behavior from being detected by others (Gross, 1998). Prior studies indicate that the affective and social outcomes of expressive suppression are different in cultures that have distinct values on emotional display during social interaction (Butler et al., 2007; Matsumoto et al., 2008; Yuan et al., 2015b). Habitually, people tend to more frequently use suppression in collectivistic cultures (e.g., Chinese culture) that highlight the avoidance of hurting others, and the efforts to preserve and experience relational harmony, than in cultures that emphasize the promotion and protection of people’s independent pursuit of positive experiences (Matsumoto et al., 2008). Affectively, our recent study in a Chinese sample found that expressive suppression decreased subjective experience of negative emotions as effectively as reappraisal (Yuan et al., 2015b). Socially, many of suppression’s negative social impacts, such as less social closeness and support, were found to be reduced when individuals with more Asian values (Butler et al., 2007).

Given that collectivistic culture values emotional suppression, it seems plausible that Chinese individuals with higher self-reported habitual use of expressive suppression are more likely to show lower neural activity within the emotion-generative regions (e.g., amygdala) during emotion induction. However, the cognitive costs of expressive suppression remain stable across cultures. An early study has suggested that expressive suppression produces great cognitive consequences, such as impaired incidental memory for information presented during the suppression period (Richards and Gross, 1999). One of our recent study in a Chinese sample has also demonstrated that though expressive suppression decreased subjective experience of negative emotions as effectively as reappraisal, expressive suppression induced larger amplitudes compared to reappraisal in central-frontal P3, a component established to reflect response inhibitory processing during behavioral inhibition studies (Yuan et al., 2015b). That is, the cognitive costs of emotion regulation by expressive suppression are greater than by cognitive reappraisal, not matter whether East Asian or Western culture is concerned (Richards and Gross, 2000). Further, Drabant et al. (2009) have suggested that spontaneous reappraisal still requires some efforts, as evidenced by the increasing control-related cortical activations (e.g., dorsal lateral PFC) with greater use of reappraisal. Therefore, it is reasonable that spontaneous suppression taxes more cognitive resources than spontaneous reappraisal, and thus people are likely not to spontaneously suppress their emotions because of its relatively higher cognitive costs.

To decrease the relatively high cognitive costs of expressive suppression, the present study employed a synonym matching task (Yang et al., 2015) to prime participants with spontaneous (automatic) expressive suppression goals, mainly because previous research has suggested that the non-conscious or automatic goal pursuit can occur without subjective awareness, and thereby consume little or no psychological and physiological cost (Mauss et al., 2007; Koole and Rothermund, 2011; Yuan et al., 2015a). We propose that such non-conscious goal pursuit can augment the human capacity for spontaneous expressive suppression. Given that the affective outcomes of suppression are similar with reappraisal in Chinese culture, we hypothesize that in emotion suppression priming condition, but not during simple emotion induction, participants with higher reported use of suppression should be more likely to engage in spontaneous suppression and should thus show decreased emotional responses at the emotion-generative region amygdala. We concentrate on amygdala activity because previous studies of both explicit and implicit emotion regulation have indicated that amygdala activity represent key neural underpinnings of negative emotion arousal and regulation (Schaefer et al., 2002; Phelps and LeDoux, 2005; Phillips et al., 2008; Etkin et al., 2010). It has also been reported that amygdala responses to negative emotional facial stimuli were associated with the habitual use of cognitive reappraisal, suggesting that individual differences in the habitual emotion regulation can be reflected by neural activity within amygdala (Drabant et al., 2009).

## Materials and Methods

### Participants

Twenty-three right-handed, healthy college students participated in the study (13 females; average age: *M* = 20.91, *SD* = 1.73). All participants gave informed consent and were paid for their participation. All participants were informed that their participation was completely voluntary and that they may withdraw from the study at any time. All participants were over 18 years of age. All participants had normal or corrected to normal vision, were right-handed, had no history of attention deficit or learning disabilities. This study was approved by the local ethical committee of Southwest University and the Institutional Human Participants Review Board of the Southwest University Imaging Center for human brain research. The experimental procedure was in accordance with the ethical principles of the 1964 Declaration of Helsinki.

### Individual Difference Measures

Individual difference measures were administered before fMRI scanning. The primary measure of interest was the suppression scale of the ERQ (Gross and John, 2003). This scale consists of four items designed to assess individual differences in suppression use (e.g., “I control my emotions by not expressing them”). This scale previously has been shown to have good internal consistency and test-retest reliability and to be independent of intelligence and socioeconomic status (Gross and John, 2003). Suppression was normally distributed according to the Shapiro–Wilk test (*p* > 0.05). Control measures were also administered including: (1) the Chinese-version of 48-item Neuroticism questionnaire of the NEO Five Factor Personality Inventory (Costa and MacCrae, 1992), which assesses an individual’s tendency to experience psychological distress; (2) the trait version of the State Trait Anxiety Inventory (STAI trait version; Spielberger, 1970), which assesses relatively stable individual differences in anxiety proneness; and (3) the reappraisal scale of the ERQ, which assesses use of cognitive reappraisal in everyday life.

### Emotion Induction Paradigm

A classic instructed fear paradigm was used as this paradigm has been verified to evoke socially instructed fear effectively (Phelps et al., 2001, 2004; Olsson and Phelps, 2004, 2007). After subjects lay supine in an MRI scanner, electrodes were attached to their left wrist. We then tested the maximum intensity of shock that participants can stand used by the electric shock equipment. Participants were informed that (1) two types of stimuli representing the two trial types would be presented: a blue square and a yellow square; (2) they might receive such a shock from the electrode attached to their wrist when one of the colored squares were presented (the threat condition), but not when the other colored square was presented (the safe condition); (3) there would be between one and three shocks delivered throughout the study. The colors representing threat and safe were counterbalanced across subjects. This process was done to convince participants that such shocks may occur during the scanning, whereas neither the threat nor safe condition was actually paired with a shock throughout the experiment. Such fear acquired by learning through verbal instruction without actually experiencing electric shocks is referred to as instructed fear (Mechias et al., 2010).

### The Synonym Matching Task and Materials

In order to activate the unconscious suppression goal, we used a synonym matching task including 54 Chinese four-character idioms. This task had been verified to prime unconscious emotion regulation successfully (Yang et al., 2015). In the matching task, participants saw a target idiom at the bottom of the computer screen and two probe idioms at the left and right side of the top of the screen. Subjects had 4 s to indicate which one of the two probe idioms was the synonym of the target idiom by pressing buttons (1 = left and 2 = right). Half of the matching idioms were presented on the right side of the screen and half of them were presented on the left side.

The 54 Chinese four-character idioms were classified into two categories according to their meaning, i.e., emotion suppression and neutral idioms. The emotion suppression idioms include 12 idioms that were selected from popular modern Chinese sayings, and these idioms advise people to keep calm in face of any consequence (e.g., ‘[scale=.50]img001’, which means keeping calm and cool in an emergency). The neutral concepts are uncorrelated to emotion regulation (e.g., ‘[scale=.50]img002’, which means objective and impartial). These idioms were not repeated within the experiment to avoid habituation.

The extent to which all the 54 idioms related to expressive suppression behavior was evaluated using an 8-point scale (0 = no correlation, 7 = high correlation) by an independent sample consisting 21 college students (14 females, mean age 24 ± 2.1). We compared the scores of the 12 idioms that belonged to the category of expressive suppression with the mean of the 42 neutral idioms. Results showed that the scores of the 12 idioms were significantly higher than the mean of neutral idioms (5.8 vs. 2.8, *t* = 9.8, *p* < 0.0001), confirming the 12 idioms were more related to expressive suppression than the neutral idioms. Besides, these idioms were also evaluated using a 9-point scale for valence (1 = extremely negative to 5 = neutral to 9 = extremely positive), arousal (1 = extremely calm to 5 = neutral to 9 = exciting), and familiarity (1 = extremely unfamiliar to 5 = neutral to 9 = extremely familiar) dimensions. A paired-samples *t*-test revealed that there were not significantly differences between the emotion regulation and the neutral idioms in the three dimensions [valence: 6.40 vs. 5.87, *t*(52) = 1.35, *p* = 0.184; arousal: 5.74 vs. 5.82, *t*(52) = -0.46, *p* = 0.645; familiarity: 7.45 vs. 7.51, *t*(52) = -0.68, *p* = 0.496].

### fMRI Design

**FIGURE 1:**
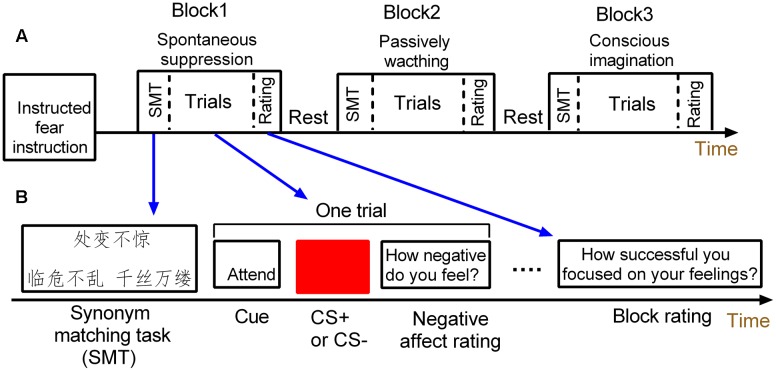
**Schematic of the mixed design.** **(A)** The mixed design has three task blocks. **(B)** Each task block consists of a synonym matching task, four threat and four safety trials and one block rating.

A mixed fMRI design (Visscher et al., 2003) was used to induce instructed fear and to assess the signal changes in amygdala activity across the conditions of passively viewing and spontaneous suppression (**Figure 1**). The experimental paradigm includes three 8-min sessions. Each session consisted of three task blocks: two emotion regulation blocks (conscious and spontaneous emotion regulation conditions) and one watching block. The task blocks were intermixed and presented randomly across sessions. Each block included four threat and four safety trials. Trial order within each block was pseudorandomized. This experiment consisted of 72 trials.

Each block started with a 4 s synonym matching task to prime participants with either a suppression goal or neutral concepts. Specifically, the spontaneous suppression block was always paired with a goal of emotion suppression by implicitly priming subjects with suppression-related meanings, while the blocks of conscious emotion regulation and watching conditions were paired with neutral concepts.

During each block, each trial was composed of three parts. First, a cue word (attend or imagine) appeared centrally for 2 s. In the block of conscious emotion regulation, the “imagine” cue was presented and subjects were asked to were asked to try to imagine something in nature which was calming when viewing the conditioned stimulus (CS). For example, participants could think of an image of the ocean or a blue sky when viewing the blue square, or square they could think canola flower fields. In the spontaneous suppression and watching blocks, the “attend” cue instructed the participant to view the stimulus and attend to their natural feelings regarding the type of the presented CS. Second, a blue or yellow square was then presented centrally for 4 s. One of the colored square (e.g., blue) was paired with the unconditioned stimulus (US; the electric shock), thus serving as the CS+, while the other square (e.g., yellow) served as the control stimulus (CS–). Neither CS+ nor CS- was actually paired with a shock throughout the experiment. The inter-trial interval (ITI) varied among 6, 8, and 10 s. The trial ended when participants were required to rate the extent of experienced fear on a 4 s 7-point scale (“how negative do you feel”; 1 = not at all; 7 = extremely). Each block ended with a 4 s rating to assess how concentrate or how success in imagination when square shown, including “how successful you focused on your feelings?” or “how successful you imagined something?”

### fMRI Acquisition and Analysis

Brain images were acquired with a Siemens 3T scanner (Siemens Magnetom Trio TIM, Erlangen, Germany). Anatomical images were collected with a T1-weighted protocol (TR = 1900 ms, TE = 2.52 ms, FA = 9°, matrix = 64 × 64, FoV = 256 × 256 mm^2^, voxel size = 1 × 1 × 1 mm^3^). The functional MRI images were collected with an Echo-Planar imaging (EPI) sequence (TR = 2 s, TE = 30 ms, flip angle = 75°, matrix size = 64 × 64, FoV = 220 × 220 mm^2^, voxel size = 3.4 × 3.4 × 3 mm^3^, Slices = 32). Before the scanning, all subjects were suggested to motion as little as possible in the experiment. Stimulus presentation and behavioral data acquisition were obtained by E-prime software.

Each functional run was subjected to preprocessing steps using DPABI (Chao-Gan and Yu-Feng, 2010) software: slice-timing, realignment, normalizing to MNI space using the structure information from coregistration and segmentation and spatial smoothing with a Gaussian kernel (8 mm FWHM).

The statistical analysis of the preprocessed functional data was performed statistical parametric mapping (SPM8^1^), and custom-written programs in Matlab. In the first-level analysis, the three functional scanning runs were modeled in one general linear model (GLM). Four periods of interest (attend-CS+, attend-CS-, unconscious-CS+, unconscious-CS-) were included in the model to compute for linear contrast maps. Six realignment parameters were further included as regressors of no interest to account for head motion effects. The resulted design matrix was then filtered with a high-band pass of 128 s.

A region-of-interest (ROI) analysis was next conducted. Given our *priori* hypotheses regarding the relationship between suppression scores and amygdala activity, masks for amygdala were applied bilaterally based on Anatomical Automatic Labeling (Tzourio-Mazoyer et al., 2002). Percent signal change (PSC) for amygdala was then extracted using MarsBaR (Brett et al., 2002). To test the hypothesis, two kinds of average contrast values for the amygdala voxels were computed. First, the emotional outcomes during the passively viewing (watching block) were represented by the contrast threat vs. safety, and were examined statistically using paired *t*-test. Second, because we were more interested in the changes of amygdala activity during spontaneous suppression, the regulatory effects of spontaneous suppression on amygdala were represented as the contrast between threat-watching condition with suppression priming and threat-watching condition without suppression priming. Correlations analysis was then conducted among these emotional outcomes and the habitual use of expressive suppression.

## Results

**FIGURE 2:**
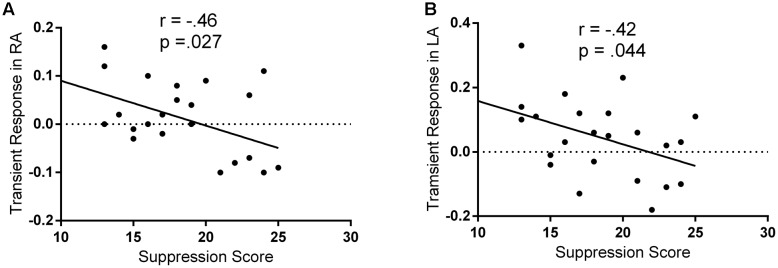
**Negative correlations between suppression and fear-related amygdala activity.** The regulatory effects of spontaneous suppression on fear-related amygdala activity were represented as the contrast between threat-watching condition with suppression priming and threat-watching condition without suppression priming. **(A)** Scatter plot of ERQ suppression and the regulatory effects of spontaneous suppression in right amygdala (RA). **(B)** Scatter plot of ERQ suppression and the regulatory effects of spontaneous suppression in left amygdala (LA).

A previous fMRI study has reported that individuals with higher self-reported reappraisal scores showed lesser activation in both the left and right amygdala, suggesting that the individual differences in the implicit processing of emotion may be reflected on bilateral amygdala (Drabant et al., 2009). Therefore, Pearson correlation coefficients were then computed to assess the relationship between the suppression score and the regulatory effects of the spontaneous suppression on the bilateral amygdala. As hypothesized, there were no significant correlations between suppression scores and BOLD signal changes in amygdala during passively viewing negative stimuli (without suppression priming; all *p* > 0.05). Only in suppression priming condition, we observed significant negative correlations between the suppression scores and the regulatory effects of the spontaneous suppression on the left (*r* = -42, *p* = 0.044) and right (*r* = -0.46, *p* = 0.027) amygdala (**Figure 2**). These results suggested that only in suppression priming condition, individuals who reported using expressive suppression more frequently in everyday life showed less amygdala activation in response to negative stimuli.

Moreover, in order to identify our findings specific to suppression, stepwise linear regressions were conducted to test whether the relationship between suppression scores and amygdala activity withstood correction for neuroticism, reappraisal score and STAI. In the bilateral amygdala, the addition of neuroticism, reappraisal score and STAI did not change the model (all *p* > 0.15). Besides, no significant correlations were found between suppression scores and any of the other three variables (all *p* > 0.165). These findings indicate that our results are specific to spontaneous suppression.

## Discussion

In this fMRI study, we used a synonym matching task to prime participants with spontaneous suppression goal. We examined whether after activating suppression goal, individual differences in the tendency to use suppression would manifest in decreased amygdala responses. We also controlled the individual differences in emotion reactivity was controlled by assessing and taking into account neuroticism, trait anxiety and the habitual use of cognitive reappraisal.

Our main findings revealed that individual differences in expressive suppression did not alter fear-related neural activation during suppression-irrelevant context, and only in emotion suppression priming condition, self-reported habitual use of suppression predicted lesser bilateral amygdala activation in response to negative stimuli. Given that expressive suppression can downregulate negative emotions as effectively as cognitive reappraisal in Chinese cultures (Yuan et al., 2015b), our findings are consistent with a recent fMRI study in Western cultures, reporting self-reported reappraisal use predicted lesser amygdala activation (Drabant et al., 2009). Moreover, these effects withstood controls for emotional reactivity (as assessed by neuroticism and trait anxiety) and for the habitual use of reappraisal. Besides, together with previous findings of habitual use of reappraisal (Drabant et al., 2009), our findings confirm that individual differences in the habitual use of emotion regulation can be reflected in both the right and left amygdala.

Importantly, these findings mainly suggest that though Chinese culture values emotion suppression, the regulatory effects of spontaneous suppression depends on the situational and personal factors (Gyurak et al., 2011). That is, only in the unconscious suppression priming condition that facilitates the use of expressive suppression, individuals who reported to use expressive suppression more frequently in everyday life would show less bilateral amygdala activity during fear emotion processing. One explanation of our findings is that emotion regulation, like any other motivated behavior, can be thought to occur as a joint function of its costs and its benefits (Richards and Gross, 1999, 2000). Without unconsciously priming suppression goals, the cognitive costs of spontaneous suppression may be higher than the benefits from doing so. However, in suppression priming condition, the costs of using spontaneous suppression may decrease and the possibility of using spontaneous suppression may thus increase. The explanation is partially supported by a recent study of emotion regulation choice (Suri et al., 2015). This study reported that in a laboratory decision context, reappraisals were implemented for only 16% of the available opportunities, and providing support for the creation of reappraisals marginally increased the percentage trials actually reappraised.

It was also important to note that our findings should be considered in the context of East Asian cultures. Because cross-cultural studies have suggested the many of suppression’s emotional outcomes may be moderated by cultural values (Butler et al., 2007; Soto et al., 2011). For western subjects, their habitual use of suppression is associated with a range of negative outcomes, such as higher levels of negative affect, lower levels of positive affect, worse interpersonal functioning and decreased well-being (Butler et al., 2003; Gross and John, 2003; John and Gross, 2004). On the contrary, our findings suggested that Chinese individuals’ habitual use of expressive suppression may be associated with positive emotion regulation outcomes.

However, several important limitations should be acknowledged in this study. First, only Chinese participants were studied. Because cultural differences have been reported in the studies of expressive suppression (Butler et al., 2007; Matsumoto et al., 2008; Soto et al., 2011), it is important to examine whether the regulatory effects of spontaneous suppression would be moderated by different cultural values. Second, we only examined one task context—namely, control of basic emotion fear. The ability to control fear is critical for our survival and adaptation, whereas the inability to control fear is a biomarker of post-traumatic stress disorder (PTSD; Jovanovic et al., 2010). However, it is important to go beyond basic emotion to better characterize the regulatory effects of spontaneous suppression across different contexts. Third, habitual suppression was only assessed by self-report measure (ERQ). Though previous research demonstrated that the ERQ has good internal consistency and test-retest reliability (Gross and John, 2003), it will be helpful to also employ non-self-report measures of habitual emotion regulation, such as peers-report, to look for convergence across diverse measures.

## Author Contributions

ZD, SC, and JYu designed experiments. ZD and QL carried out experiments. SC and ZD analyzed experimental results. SC and JYu wrote the manuscript. YX analyzed experimental results and wrote the manuscript. JYu and JYa supervised and revised the manuscript critically.

## Conflict of Interest Statement

The authors declare that the research was conducted in the absence of any commercial or financial relationships that could be construed as a potential conflict of interest.

## Footnotes

1 www.fil.ion.ucl.ac.uk/spm
